# Evolution of Exon-Intron Structure and Alternative Splicing

**DOI:** 10.1371/journal.pone.0018055

**Published:** 2011-03-25

**Authors:** Tomasz E. Koralewski, Konstantin V. Krutovsky

**Affiliations:** Department of Ecosystem Science and Management, Texas A&M University, College Station, Texas, United States of America; Centre de Regulació Genòmica, Spain

## Abstract

Despite significant advances in high-throughput DNA sequencing, many important species remain understudied at the genome level. In this study we addressed a question of what can be predicted about the genome-wide characteristics of less studied species, based on the genomic data from completely sequenced species. Using NCBI databases we performed a comparative genome-wide analysis of such characteristics as alternative splicing, number of genes, gene products and exons in 36 completely sequenced model species. We created statistical regression models to fit these data and applied them to loblolly pine (*Pinus taeda* L.), an example of an important species whose genome has not been completely sequenced yet. Using these models, the genome-wide characteristics, such as total number of genes and exons, can be roughly predicted based on parameters estimated from available limited genomic data, e.g. exon length and exon/gene ratio.

## Introduction

Recent advances in high-throughput DNA sequencing led to significant progress in complete genome sequencing and opened unprecedented opportunities for comparative genome studies [1]–[4]. The complete genome sequences are publicly available from constantly growing databases, such as the National Center for Biotechnology Information (NCBI) GenBank, and can be readily analyzed and compared for a number of evolutionarily distant species. The early comparisons revealed that the number of genes and metabolomic complexity progressively increase as species become more evolutionarily advanced [5]–[8], but their anatomical, morphological, physiological and behavioral complexity does not linearly correlate with the total number of genes discovered. For instance, whereas the number of protein coding genes in the human genome is only 14% greater than in the roundworm *Caenorhabditis elegans*, the evolutionary differences between these two species are immense. This suggests that regulatory and post-transcriptional processes might play an increasingly more important role throughout evolution. There are numerous mechanisms, processes and structures that affect gene regulation, such as methylation, chromatin structure, regulatory elements, transcription factors, polyadenylation, post-translational modifications and compartmentalization of proteins, and others (for review see [9]). RNA editing and alternative splicing (AS) are two distinct post-transcriptional processes that can, however, increase proteomic complexity and number of various proteins without increasing the number of genes. While RNA editing, particularly common in organelles (for review see [10], [11]), can lead to nucleotide insertion or deletion, in higher Eukaryotes base modifications are prevailing [11]–[13]. In the process of AS additional variants are created among the mature mRNA transcripts through modification and rearrangement of exons (e.g. [14], [15]). Both mechanisms promote adaptive and evolutionary potential of species without increasing the number of genes and maintenance cost that could be associated with it. For instance, due to AS the total hypothetical number of various proteins encoded by the *DSCAM* gene can reach 38,016 in *Drosophila melanogaster* [16]. Therefore, one may expect that more evolutionarily advanced organisms have more elaborated and complex AS. We addressed this hypothesis in more detail in our study. Our objectives were to examine exon-intron structure in genomes of completely sequenced and fully annotated species, to infer AS data and to use this information for defining relationships between genes and proteomic complexity. We expect that these relationships can be used to predict the anticipated exon-intron structure and proteomic complexity in non-model species with large genomes, such as pines, that may remain unsequenced for a while. We applied our findings to loblolly pine (*Pinus taeda* L.), one of the most-studied coniferous species, which has a very large genome of 24.56 pg (∼24 Gb) [17]; complete genome sequencing for loblolly pine is underway [18], but is still problematic and unavailable. The obtained knowledge is also essential for understanding the genetic control of the metabolomic complexity and functionality in the studied species and the evolutionary significance of AS in general.

## Results

### Analysis of complete genomes

**Figure 1:**
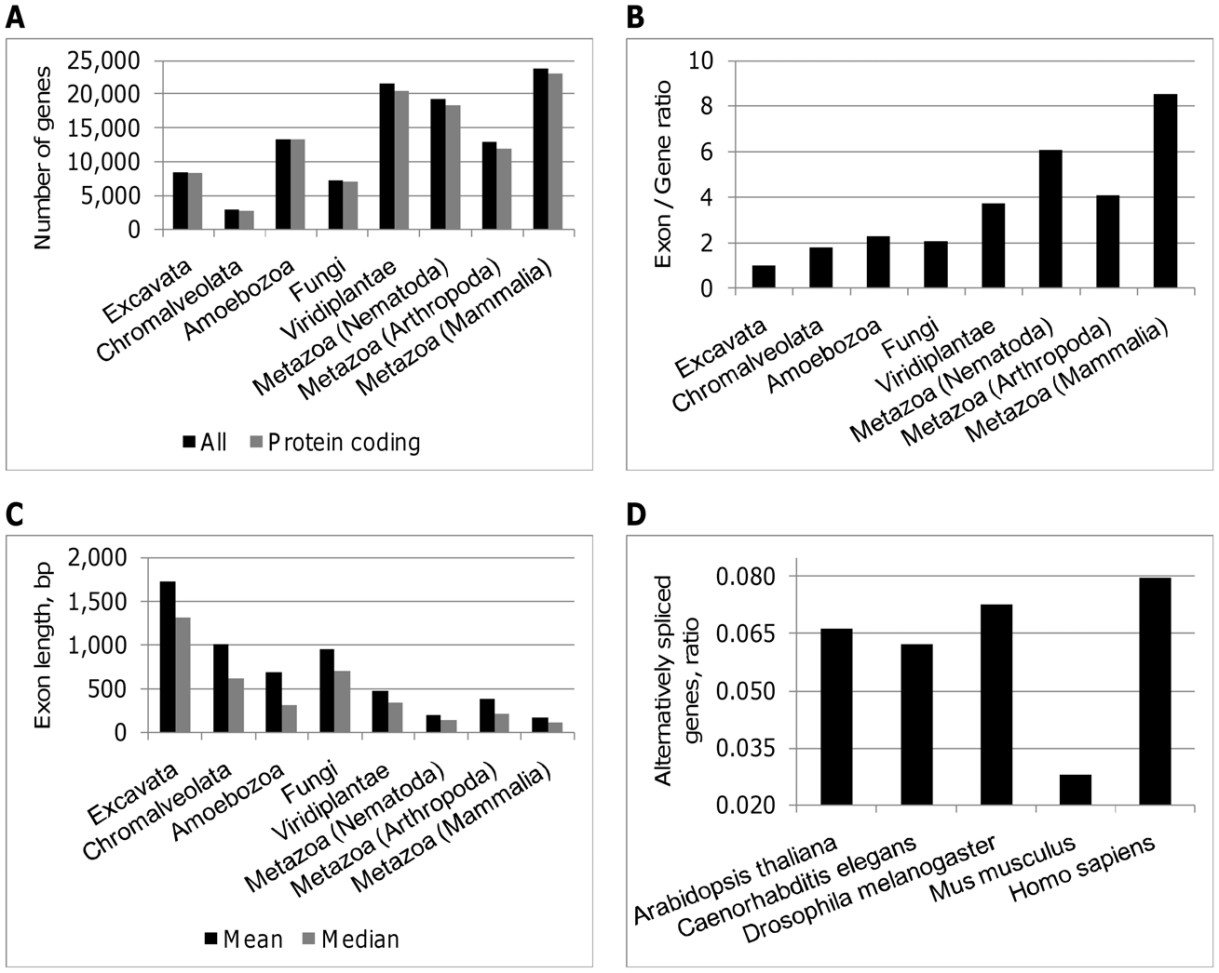
Comparison of selected genome parameters across taxonomic groups (A–C) and species (D). Number of all (black) and protein coding (gray) genes (**A**), exon/gene ratio (**B**), and mean (black) and median (gray) exon lengths (**C**) averaged over taxonomic groups, and ratio of alternatively spliced genes in five species (**D**).

First, to explore general trends in the completely sequenced genomes, we analyzed basic statistics (Table 1 and Table S1). Previously Lynch and Conery [6] showed strong positive correlation between genome size and gene number in multiple species. Here, we observed an increase in the number of genes (Fig. 1A), gene products and total number of exons as species advance evolutionarily. The exon/gene ratio also increases (Fig. 1B), but the mean and median exon length becomes shorter (Fig. 1C), whereas CDS length remains relatively constant. Because the changes in parameter values demonstrated clear trends that followed evolutionary advancement, we proceeded with an in-depth correlation analysis. The results of regression analysis and estimates of the parameters are summarized in Table 2 and Table S2.

**Table 1:** Exon-intron gene structure in completely sequenced genomes of 36 species.

Taxonomic group	Species	All genes	Protein coding genes	Protein coding/All gene ratio	CDSs	Exons	Mean exon length	Mean CDS length	Exon/Gene ratio
Excavata	*Leishmania braziliensis*	7,898	7,897	0.9999	7,898	7,998	1,844.8	1,868.2	1.013
	*Leishmania infantum*	7,993	7,993	1.0000	7,993	8,069	1,839.9	1,857.6	1.010
	*Trypanosoma brucei*	9,336	8,772	0.9396	8,772	8,774	1,506.2	1,506.5	1.000
Chromalveolata	*Cryptosporidium parvum*	3,885	3,396	0.8741	3,396	3,440	1,821.1	1,844.7	1.013
	*Guillardia theta*	742	632	0.8518	632	648	851.1	872.6	1.025
	*Hemiselmis andersenii*	524	471	0.8989	471	471	1,018.3	1,018.3	1.000
	*Plasmodium falciparum*	5,300	5,263	0.9930	5,267	12,651	949.6	2,280.8	2.404
	*Theileria parva*	4,089	4,035	0.9868	4,035	14,447	393.2	1,408.0	3.580
Amoebozoa	*Dictyostelium discoideum*	13,322	13,322	1.0000	13,331	30,441	686.9	1,569.2	2.285
Fungi	*Aspergillus fumigatus*	9,859	9,630	0.9768	9,630	28,259	504.0	1,479.0	2.934
	*Aspergillus niger*	14,420	14,086	0.9768	14,086	50,371	370.4	1,324.4	3.576
	*Candida glabrata*	5,499	5,271	0.9585	5,272	5,356	1,485.5	1,509.2	1.016
	*Cryptococcus neoformans*	6,407	6,273	0.9791	6,475	39,350	257.2	1,608.8	6.273
	*Debaryomyces hansenii*	7,081	6,866	0.9696	6,872	7,227	1,274.3	1,340.5	1.053
	*Encephalitozoon cuniculi* *	2,029	1,996	0.9837	1,996	2,011	1,072.3	1,080.4	1.008
	*Eremothecium gossypii* *	4,971	4,714	0.9483	4,714	4,940	1,406.1	1,474.7	1.048
	*Gibberella zeae*	11,619	11,619	1.0000	11,619	37,454	477.2	1,538.2	3.224
	*Kluyveromyces lactis*	5,504	5,331	0.9686	5,331	5,461	1,377.1	1,410.7	1.024
	*Neurospora crassa*	10,093	9,699	0.9610	9,709	26,598	533.5	1,462.6	2.742
	*Pichia stipitis*	5,816	5,816	1.0000	5,816	8,383	1,025.5	1,478.1	1.441
	*Saccharomyces cerevisiae* *	6,136	5,861	0.9552	5,861	6,185	1,412.6	1,490.7	1.055
	*Schizosaccharomyces pombe* *	5,374	5,083	0.9459	5,084	9,844	722.9	1,400.4	1.937
	*Ustilago maydis*	6,604	6,495	0.9835	6,495	11,373	1,052.2	1,842.4	1.751
	*Yarrowia lipolytica*	7,180	6,660	0.9276	6,661	7,402	1,295.4	1,439.7	1.111
Viridiplantae	*Arabidopsis thaliana* *	28,245	26,977	0.9551	30,705	138,876	236.8	1,208.0	5.148
	*Oryza sativa* *	29,102	26,777	0.9201	26,777	128,267	250.2	1,198.3	4.790
	*Ostreococcus ‘lucimarinus’*	7,603	7,603	1.0000	7,603	9,767	944.8	1,213.7	1.285
Metazoa	*Caenorhabditis briggsae*	17,363	16,429	0.9462	16,429	98,457	209.8	1,257.1	5.993
(Nematoda)	*Caenorhabditis elegans* *	21,172	20,174	0.9529	23,759	124,949	203.1	1,322.0	6.194
Metazoa	*Anopheles gambiae*	12,423	11,971	0.9636	12,500	48,875	358.2	1,454.4	4.083
(Arthropoda)	*Drosophila melanogaster* *	14,807	13,887	0.9379	17,837	56,580	401.0	1,719.8	4.074
	*Drosophila pseudoobscura*	11,875	9,606	0.8089	9,707	39,256	383.9	1,553.0	4.087
Metazoa	*Canis lupus familiaris*	19,384	19,380	0.9998	31,837	194,624	169.2	1,748.3	10.043
(Mammalia)	*Homo sapiens* *	25,074	23,055	0.9195	27,904	201,083	174.5	1,548.4	8.722
	*Mus musculus* *	26,314	25,533	0.9703	27,159	200,714	179.4	1,420.9	7.861
	*Pan troglodytes*	23,962	23,881	0.9966	40,767	177,922	170.2	1,440.1	7.450

*The most annotated species (see Materials and Methods for details).

**Table 2:** Predicted values for exon-intron gene structure and alternative splicing (AS) parameters for an organism with mean and median exon lengths of 334.8 and 198.0 bp, respectively, such as observed in *Pinus taeda*, based on results of regression analysis.

Response (*y*)	Factor (*x*)	*R* ^2^	*R* ^2^_adj_	*P*-value at 95% CI	Figure	Predicted	95% CI at population level		95% CI at individual level	
							(lower/upper)		(lower/upper)	
Number of exons	Mean exon length	0.937	0.936	<0.0001	2A	53,374	47,887	58,860	20,093	86,655
Number of protein coding genes	Mean exon length	0.712	0.703	<0.0001	2B	13,288	11,780	14,797	4,824	21,752
Number of all genes	Mean exon length	0.706	0.697	<0.0001	S1	13,871	12,270	15,471	4,891	22,850
Exon/Gene ratio	Mean exon length	0.957	0.956	<0.0001	2C	4.245	4.049	4.441	3.146	5.344
Mean CDS length	Mean exon length	0.065	0.038	0.1321	S2	-				
Number of protein coding genes	Number of exons	0.897	0.894	<0.0001	2D	-				
Number of all genes	Number of exons	0.891	0.888	<0.0001	S3	-				
Number of protein coding genes	Number of all genes	0.996	0.996	<0.0001	2E	-				
Number of exons	Exon/Gene ratio	0.864	0.860	<0.0001	2F	-				
Number of protein coding genes	Exon/Gene ratio	0.648	0.638	<0.0001	S4	-				
AS	Mean exon length	0.615	0.576	0.0025	S5	0.018	0	0.053	0	0.117
AS	Exon/Gene ratio	0.498	0.448	0.0103	S6	-				
AS	Number of CDSs	0.725	0.698	0.0004	S7	-				
Number of exons	Mean exon length	0.999	0.998	0.0175	-	71,010*	49,220	92,801	29,385	112,636
Number of protein coding genes	Mean exon length	0.997	0.994	0.0351	-	19,785*	13,411	26,159	7,063	32,508
Number of all genes	Mean exon length	0.990	0.980	0.0632	-	20,923*	8,371	33,474	0	45,975
Number of exons	Median exon length	0.852	0.848	<0.0001	-	59,904	51,343	68,465	8,737	111,070
Number of protein coding genes	Median exon length	0.715	0.707	<0.0001	-	11,827	10,422	13,231	3,432	20,221
Number of all genes	Median exon length	0.710	0.701	<0.0001	-	12,342	10,853	13,831	3,441	21,243
Exon/Gene ratio	Median exon length	0.903	0.901	<0.0001	-	3.658	3.382	3.934	2.007	5.308
Median CDS length	Median exon length	0.035	0.006	0.2767	-	-				

*Models constructed based on three plant species; see text for details.

### Average exon length as a predictor

**Figure 2:**
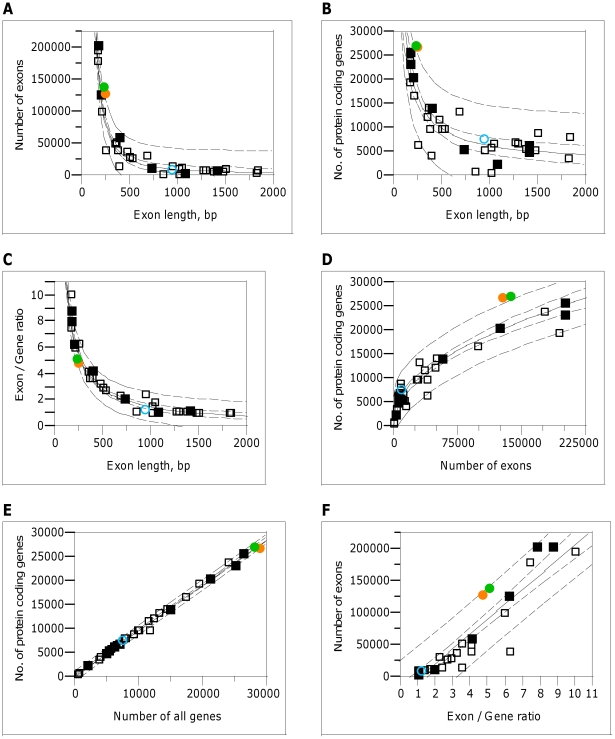
Correlations between selected genome parameters. Correlations of number of exons and mean exon length (**A**), number of protein coding genes and mean exon length (**B**), exon/gene ratio and mean exon length (**C**), number of protein coding genes and number of exons (**D**), number of protein coding genes and number of all genes (**E**), and number of exons and exon/gene ratio (**F**) based on 36 species studied. The most annotated 10 species are represented by solid markers. Green, orange and blue markers correspond to *Arabidopsis thaliana*, *Oryza sativa* and *Ostreococcus ‘lucimarinus’*, respectively. 95% confidence intervals are presented for both population (internal dashed line) and individual (external dashed line) levels.

Generally, the length of exon can be approximated from a limited sample of genes. Hence, we explored its potential for predicting other genomic parameters in incompletely sequenced species. A very strong negative correlation was observed between mean exon length and total number of exons (*r* ^2^ = 0.937, *r* ^2^_adj_ = 0.936; Fig. 2A). The negative correlation was weaker but statistically significant between mean exon length and either number of protein coding genes (*r* ^2^ = 0.712, *r* ^2^_adj_ = 0.703; Fig. 2B) or the total number of genes (*r* ^2^ = 0.706, *r* ^2^_adj_ = 0.697, Fig. S1). Mean exon length also strongly negatively correlated with exon/gene ratio (*r* ^2^ = 0.957, *r* ^2^_adj_ = 0.956; Fig. 2C). In all these cases the correlations were not linear. No statistically significant correlation was observed between mean exon length and mean CDS length (*P* = 0.132; Fig. S2). These results proved that exon length is, indeed, a robust predictor.

Similar correlations were observed for median exon length, and the *r* ^2^ values were close to those obtained for mean exon length (see Table 2 for details).

### Number of genes and exons in genomes

Since both number of genes and number of exons increase with the species complexity, we measured how robust the correlation between these parameters was. Strong and nonlinear but positive correlations were found between the number of genes and number of exons (*r* ^2^ = 0.897, *r* ^2^_adj_ = 0.894 for protein coding genes, Fig. 2D; *r* ^2^ = 0.891, *r* ^2^_adj_ = 0.888 for the total number of genes, Fig. S3). Also very strong positive correlation was observed between the total number of genes and number of protein coding genes (*r* ^2^ = 0.996, *r* ^2^_adj_ = 0.996; Fig. 2E). These tight relationships show clear directions in the genome evolution, where not only amount of genetic information is increasing but also is accompanied by fragmentation that facilitates its dynamic use. The linearity and extremely high *r^2^* value between all genes and protein coding genes also shows that the non-protein coding gene fraction changes proportionally.

### Exon/gene ratio as a predictor

Similarly as the exon length, exon/gene ratio has a high predictive power for complex genomic parameters. Strong positive linear correlation was observed between exon/gene ratio and the total number of exons in the genome (*r* ^2^ = 0.864, *r* ^2^_adj_ = 0.860; Fig. 2F). The relationship between exon/gene ratio and the number of protein coding genes was also strongly positive but rather nonlinear (*r* ^2^ = 0.648, *r* ^2^_adj_ = 0.638; Fig. S4).

### Alternative splicing

The traditional methods of using EST data to study AS are sensitive to the EST coverage [19]. To diminish biases related to this problem we used the NCBI GenBank annotations of the well-annotated genomic data for 12 model species in our study. Despite numerous AS studies (see Discussion) we are unaware of any that would take an advantage of thorough annotations. The five most annotated species (*A. thaliana*, *C. elegans*, *D. melanogaster*, *M. musculus* and *H. sapiens*) were analyzed in more detail (Table 3). Among the five most common AS types, alternative 3′ splice sites type (A3) was the most frequent in *A. thaliana* and *C. elegans*, but exon skipping (ES) was the most frequent in *D. melanogaster*, *M. musculus* and *H. sapiens*.

**Table 3:** Alternative splicing types observed in five most studied species.

Species	ES	IR	A3	A5	ME	A5A3	ESA3	A5ES	MEA3	A5ME	ESES	A5ESA3	Other	N	A	R	*H*	*H_max_*	*E*
*Arabidopsis thaliana*	106	428	960	396	4	195	11	9	0	0	15	10	34	1,787	0.066	1.138	7.38	7.49	0.985
*Pr*	*0.049*	*0.197*	*0.443*	*0.183*	*0.002*	*0.090*	*0.005*	*0.004*	*0.000*	*0.000*	*0.007*	*0.005*	*0.016*						
*Caenorhabditis elegans*	407	194	527	318	34	59	9	12	1	0	104	18	86	1,251	0.062	1.177	6.95	7.13	0.975
*Pr*	*0.230*	*0.110*	*0.298*	*0.180*	*0.019*	*0.033*	*0.005*	*0.007*	*0.001*	*0.000*	*0.059*	*0.010*	*0.049*						
*Drosophila melanogaster*	464	292	297	165	102	62	31	16	2	0	79	9	67	1,008	0.073	1.284	6.66	6.92	0.964
*Pr*	*0.293*	*0.184*	*0.187*	*0.104*	*0.064*	*0.039*	*0.020*	*0.010*	*0.001*	*0.000*	*0.050*	*0.006*	*0.042*						
*Mus musculus*	423	128	133	72	35	32	18	5	5	1	126	5	41	714	0.028	1.064	6.40	6.57	0.974
*Pr*	*0.413*	*0.125*	*0.130*	*0.070*	*0.034*	*0.031*	*0.018*	*0.005*	*0.005*	*0.001*	*0.123*	*0.005*	*0.040*						
*Homo sapiens*	1,519	92	468	262	124	41	25	19	2	0	294	20	114	1,834	0.080	1.210	7.26	7.51	0.966
*Pr*	*0.510*	*0.031*	*0.157*	*0.088*	*0.042*	*0.014*	*0.008*	*0.006*	*0.001*	*0.000*	*0.099*	*0.007*	*0.038*						

ES – exon skipping; IR – intron retention; A3 – alternative 3′ splice site; A5 – alternative 5′ splice site; ME – mutually exclusive exons; N – number of alternatively spliced genes; A – alternative splicing ratio (proportion of alternatively spliced genes); R – ratio of the total number of protein products to the total number of protein genes; *H* – Shannon's index; *H_max_* – maximum possible value of Shannon's index, where for a given *n*, *H* is a maximum and equal to log*n*, when all the *P*i are equal (i.e., 1/*n*);*E* – Shannon's equitability (*E* = *H*/*H_max_*). Complex cases are denoted as combinations of these abbreviations. *Pr* - proportion (ratio) of the type to all types.

**Figure 3:**
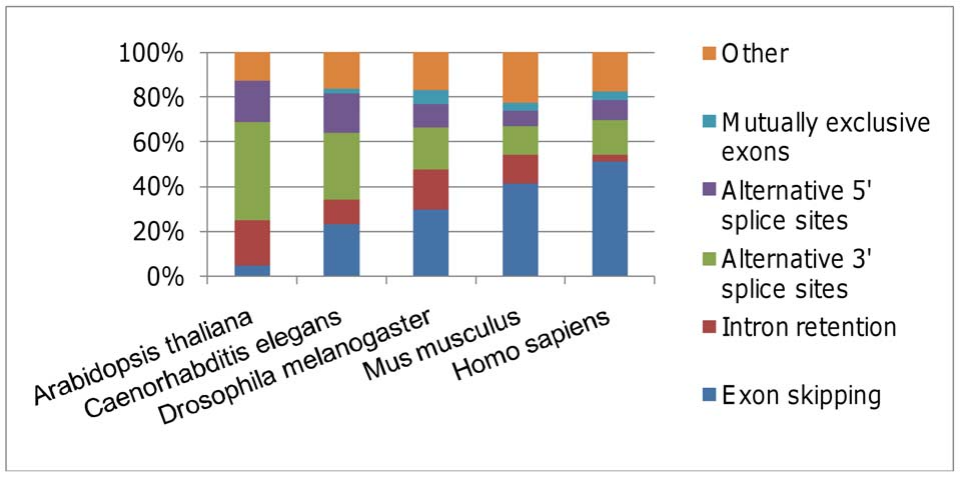
Relative frequency of alternative splicing (AS) types in five species. The category “Other” includes complex schemes of AS, e.g. simultaneous occurrences of alternative 5′ and 3′ splice sites within an intron.

The frequency of the ES type increases following organism complexity and reaches more than 51% of all AS types in human (Fig. 3). It is accompanied by a decrease in the intron retention (IR), A3 and alternative 5′ splice sites (A5) types. In the plant model, *A. thaliana*, the most common type is A3 (44.3%), followed by IR (19.7%).

### Alternative splicing ratio

The AS ratio, that is the ratio of the alternatively spliced genes and the total number of protein coding genes was highest (0.186) in *Pan troglodytes* among all analyzed species. When only the most-annotated species were considered, the highest AS ratio was observed in human (0.080), followed by the one in *D. melanogaster* (0.073) and *A. thaliana* (0.066; Table 3). In general, the ratio increases with evolutionary progress (Fig. 1D). Although these estimates seem low as compared to other AS studies (see Discussion for details), we may expect a rapid growth in the data abundance in the near future due to the fast development of novel genomic techniques (e.g. [20]), which will help fully understand the effects of this mechanism.

AS negatively correlated with exon length and occurred more frequently in organisms with shorter exons (Fig. S5; *r* ^2^ = 0.615). Similarly, AS increases as exon/gene ratio (*r* ^2^ = 0.498; Fig. S6) and the total number of CDSs (*r* ^2^ = 0.725; Fig. S7) increase.

Among the five most-annotated species, both Shannon's diversity index and equitability were highest in *A. thaliana* (*H* = 7.38, *E* = 0.985; Table 3) showing high richness and evenness of distribution. Second high value (*H* = 7.26) was observed in human, but the evenness was lower (*E* = 0.966).

### Predictions for other species with large genomes such as *Pinus taeda*

Having defined regression equations between the key genomic parameters, we applied these models to *P. taeda*, an important but incompletely sequenced species. Based on the 99 complete CDSs available in GenBank, the mean and median transcript lengths were practically the same (1278 bp; Table 4). However, the mean and median exon lengths were very different – 334.8 and 198 bp, respectively. These estimates are very preliminary and based only on 21 exons. An additional 43 complete exons were identified in partial CDSs. Their length was shorter – 166.2 bp on average, but these estimates could be biased toward shorter exons due to the PCR-biased amplicon resequencing. It is worth noting that any species, completely or incompletely sequenced, with a known estimate of the exon length could be investigated here in place of *P. taeda*. Moreover, the accuracy of the exon length estimation for any species will increase if more data are available and if the ascertainment bias (e.g. resulting from overrepresentation of certain gene families in the sample, or from inclusion of only partially sequenced genes) is kept at the minimum. Based on the regression models created for the 36 complete genomes, we computed estimates for a hypothetical species with an average exon length such as the one observed in *P. taeda* (Table 2). The predicted exon/gene ratio was 4.245, very close to the observed 4.000 in *P. taeda* (95% CI on individual level: 3.146 to 5.344). The predicted total number of exons, genes and number of protein coding genes were 53,374 (95% CI on individual level: 20,093 to 86,655), 13,871 (95% CI on individual level: 4,891 to 22,850) and 13,288 (95% CI on individual level: 4,824 to 21,752), respectively. The estimates differed slightly when median exon length was used (see Table 2 for details).

**Table 4:** Exon and CDS lengths in *Pinus taeda* based on complete CDS sequences.

Feature	CDS	Exon
Number	99	21
Mean length, bp	1278.3	334.8
Median length, bp	1278.0	198.0
Standard deviation	667.3	296.7

## Discussion

Despite significant progress in sequencing technologies, complete genomic data are still limited for eukaryotic organisms; and, more importantly, only a few extensively studied model species have been well annotated and featured. The most abundant data have been collected for microbial, fungal and some animal genomes, while vascular plants have been understudied. Only a few species have been completely sequenced and annotated in this underrepresented group so far, such as *Arabidopsis thaliana*, *Oryza sativa*, *Vitis vinifera*, *Physcomitrella patens*, and, recently, *Populus trichocarpa*, which remains relatively poorly annotated and featured. Therefore, due to insufficient experimental data, it is very likely that not all gene transcripts and AS products have been recorded in GenBank even for the best-studied species; this can cause underestimation of AS ratios in our study. As more experimental data are collected, the situation gradually improves with every new genome build that updates the number of genes, exons, and their locations on the chromosome. AS has different types, occurs at different developmental stages and tissues, and can be affected by environmental factors [21], [22]. AS is still insufficiently studied, and, therefore, not all AS events and types are well documented in the databases. Moreover, precise inference is more difficult due to incomplete annotation, along with different stringency criteria, customary thresholds to classify true and erroneous AS events, and various gene models used in different species. To avoid these complications, we limited our analyses only to extensively studied completely sequenced genomes. However, we hope that the results obtained can be used for predictions in insufficiently studied and incompletely sequenced organisms, such as pine in our study, in which investigation of exon-intron architecture is impeded by the availability of the data.

### Alternative splicing ratio

In general, both AS and number of genes are higher in more evolved organisms (Figs. 1A, D). However, surprisingly the AS rate was not always as high in more evolutionarily advanced species as expected and did not correlate linearly with their evolutionary progress. For instance, the ratio observed in *D. melanogaster* (0.073) was higher than in *A. thaliana* (0.066), but very close to the one in human (0.080). This could suggest that a relatively small number of genes in *D. melanogaster* compared to human (14,807 vs. 25,074, respectively) is compensated by a higher AS rate that increases proteomic and metabolomic complexity. We cannot completely exclude that the discrepancies are explained by insufficient AS data, but we can conclude in general that both the number of genes and the AS rate increase in more evolutionarily advanced species. Certainly, more experimental data are needed to increase the precision of estimates and predictions of AS ratios. For instance, a number of studies demonstrated AS in rice [23]–[25], but it is not documented in rice genomic data from the NCBI GenBank database, a likely indication that annotation of the rice genome is still in progress.

A substantially higher AS rate for shorter exons found in the study (Fig. S5) is consistent with previous studies that suggested that both tandem exon duplication [26] and insertion of noncoding intron sequences [27] could promote AS. Both within-gene duplication and AS would have less drastic effect on functionality of final proteins when they both deal with mutually exclusive exons (ME) that have shorter lengths. In addition, converting a part of an intron into an exon via AS has the risk of including a stop codon. This risk is higher when alternative exon sequences are longer.

Although not as drastic, higher exon/gene ratio is also associated with a higher AS rate (Fig. S6). This can be observed from the above described correlation of exon length and AS because more exons per gene mean both shorter exons (assuming a constraint on the final gene product length) and more options for AS. Exon/gene ratio, similarly to AS, increases in evolutionarily advanced species (Figs. 1B, D). More advanced species also show higher numbers of CDSs. AS increases as number of CDSs increases (Fig. S7), playing an important role in creating higher proteomic complexity.

Previous studies reported the ES type of AS as the most frequent in mammals [28], [29]. Our results are consistent with these findings. ES accounted for 51.0% of AS in human and 41.3% in mouse. Moreover, frequency of ES increases with complexity. Kim et al. [30] used a modified approach that required the final number of ESTs in the compared organisms' genes to be the same to mitigate the bias in data availability for the studied species. They also found a high frequency of ES in mammals (∼40%) and low IR (∼10%).

It is the opposite in plants, where IR type was the most frequent (over 50%) in both rice and thale cress [24], [31]. The A3 was the second most frequent type, while ME was the least frequent type. Nagasaki et al. [28] reported that IR accounted for over 42% of AS events in thale cress and 55% in rice. Although our analysis also found a very low level of ME in thale cress (0.2%), the most abundant type was A3 (44.3%) followed by IR (19.7%). Assuming that none of the classes is underrepresented in the dataset we used, this could indicate that IR tends to be overestimated in the EST/cDNA based studies, possibly due to the highest incidence of nonsense-mediated mRNA decay (NMD)-targeted products in this class. Wang and Brendel [31] estimated that ∼43% of AS events in *Arabidopsis* are potential NMD candidates, with IR showing the highest incidence of 40–48%. Conversely, Kim et al. [30] found the rate of IR in *Arabidopsis* (∼30%) less than A3 (∼40%). In their study, ES accounted for approximately 5% of all types. McGuire et al. [25] found that in *A. thaliana* IR accounted for 38.7% of splice variants, only slightly more than A3 (36.8%) and ES (7.7%) events. These results show great sensitivity to the methods and assumptions used. McGuire et al. [25] discussed how including unspliced alignments may affect the outcomes.

### Alternative evolutionary scenarios for plants

This study demonstrates that plants and animals may have used different mechanisms and strategies for developing proteomic and metabolomic complexity. The AS rate is low in plants compared to animals, whereas the number of genes is high (Figs. 1A, D). This could indicate that animals have evolved a more efficient system of managing the genomic information that allows them to increase proteomic complexity with the same or smaller number of genes. Flowering plants could have relied primarily on duplications (from exon shifting to entire chromosome or genome duplications that are common in flowering plants) [32], duplication modifications and divergence. In contrast, large genomes in genus *Pinus* (class Coniferopsida) might be a result of retrotransposon expansion rather than polyploidy [33]. Cui et al. [32] found no evidence of recent genome duplications in *P. taeda* nor *P. pinaster*, and Grotkopp et al. [17] estimated that the genome sizes varied from 22.10 pg to 36.89 pg in pines, with the putative common ancestor's genome of 32.09 pg. Nevertheless, sporadic polyploidy has been observed in gymnosperms (for review see [34]).

In order to check how well our regression models fit the real data, we compared the values observed in the three plants in our dataset with those predicted by the models. The total numbers of exons predicted based on the observed average exon length in *Oryza sativa* and *Arabidopsis thaliana* were underestimated in both cases (predicted 92,581 and 102,910 vs. observed 128,267 and 138,876, respectively), but the observed values were only slightly greater than the 95% confidence interval upper limit at the individual level (126,092 and 136,543, respectively; Fig. 2A). The observed number of exons in the primitive alga *Ostreococcus ‘lucimarinus’* (9,767) was close to the predicted number (10,021) and fell within 95% CI.

Similarly, the observed numbers of protein coding genes (Fig. 2B) in both higher plants (26,977 in thale cress and 26,777 in rice) were only slightly higher than the upper 95% CI limit at individual level (26,282 and 25,445, respectively), and predicted values were underestimated (17,694 and 16,888, respectively). The total observed number of genes (Fig. S1) for the two species, again, fell only slightly above the 95% individual level CI (28,245>27,591 for thale cress and 29,102>26,715 for rice; predicted values: 18,480 and 17,637, respectively). In both cases the numbers observed in the alga fell within the 95% CI.

The discrepancy between Viridiplantae and other kingdoms can also be observed in the relationship between exon count and number of genes (Fig. 2D, Fig. S3), as well as exon/gene ratio and number of protein coding genes (Fig. S4). In our models the values for the two higher plants fall outside the upper 95% CI at the individual level in all these cases. Interestingly, comparison of the two evolutionarily youngest groups in kingdoms Metazoa and Viridiplantae reveals much longer exons in plants (236.8 bp in *A. thaliana* and 250.2 bp in *O. sativa*) than in mammals (ranging from 169.2 bp in *Canis lupus familiaris* to 179.4 bp in *M. musculus*), demonstrating that the processes that reduce exon length have been slower in plants. Body plan complexity is much greater in mammals than in angiosperms, and shorter mammalian exons coupled with lower number of genes could indicate greater pressure towards efficient use of gene space. The highest values of Shannon's index and equitability observed in *A. thaliana* (Table 3) indicated more even AS distribution than in four animal species, despite their higher evolutionary position. Perhaps AS is not the main mechanism in achieving the observed complexity level in plants. If AS is correlated with exon length, then longer exons in plants can imply that the pressure for greater AS rates is not as strong as in the case of higher animals. Other mechanisms, such as more frequent duplications and elevated retrotransposon activity in plants could be responsible for the high number of genes, and also greater exon lengths, through intron loss. Indeed, studies on animal species have shown a negative correlation between gene family size and AS frequency [35]–[37]. This could explain not only lower rates of AS observed in plants but also the different patterns of AS forms, potentially increasing chances of NMD, a phenomenon not very well studied in plants [23]. Conversely, building upon the theory proposed by Lynch and Conery [6], Babenko et al. [38] suggested that intron gain/loss is not a commonly ongoing process, but rather may be triggered by certain dramatic evolutionary events that lead to long-term bottlenecks. Therefore the observed differences in exon lengths could be merely due to chance of the ancestors being affected by drastic events in the past. These conclusions seem to be supported by Sammeth et al. [29], showing rather abrupt differences between invertebrates and vertebrates.

Current genomic data are insufficient to build separate robust regression models for plants. The conclusions about the total number of exons, the total number of genes and the total number of protein coding genes in *P. taeda* may therefore be biased; and the true values may be close to the upper 95% CI limit on the individual level, higher than the ones predicted by the proposed models (see below).

### Short exons promote genomic complexity

The strong relationship between exon length and total number of exons (Fig. 2A) as well as exon length and exon/gene ratio (Fig. 2C) suggest that shorter exons increase potential for AS. Indeed, a much higher ratio of AS was observed in organisms with shorter exons (Fig. S5). However, more evolutionarily advanced organisms not only have shorter exons, but also more genes (Fig. 2B, Figs. 1A, C, and Fig. S1). The presence of shorter exons increases the potential for exon shuffling along with exon duplications; and, as a complement of AS, both increase proteomic and metabolomic complexity. It is likely that both evolved simultaneously and synergistically to amplify their effects on increasing physiological, behavioral and morphological complexity of the organisms through positive feedback loop-like mechanisms.

No statistically significant correlation was found between exon length and CDS length (Fig. S2). Since 3-dimensional protein structure and binding sites determine protein functionality, the length of the coding sequence seems to be of primary importance, and therefore the variation in the transcript length may be constrained. This could suggest that in the process of evolution, partitioning of the ancestral coding sequences has been occurring rather than extension through e.g. hypothetical stacking of coding blocks together. Such a process could have stimulated splicing out duplicated exons, eventually leading to alternatively spliced forms.

At the genome level, most of the species with less than 10,000 genes had a very small number of exons (Fig. 2D and Fig. S3). Consequently, the number of exons per gene was low in these species (Fig. 2F, Figs. 1A, B and Fig. S4). These observations show a general trend of genomic complexity increasing in evolutionarily advanced species.

The shortest exons identified in some of the analyzed species (including three plants) were only 1 bp long. We did not find any peer-reviewed publications experimentally confirming this observation. In previous studies Long et al. [39] identified single base pair exons, and Deutsch and Long [40] identified exons as short as 1 amino acid in a number of species including *A. thaliana* and human, although the exact length in bp is not clear. An experimental approach is necessary to find support for these structures and to verify that it is not an artifact resulting from exon/intron model assumptions. For instance, Kondrashov and Koonin [26] used 9 bp as threshold.

### Implications for *Pinus taeda*

Due to the small sample size, the *P. taeda* exon length estimate may be significantly biased. An alternative would be to include in the study completely sequenced exons from only partially sequenced genes. However, in this scenario shorter exons would be overrepresented due to the PCR amplicon length bias (typically a few hundred bp), making the mean and median underestimated. The observed exon/gene ratio was 4.000 based on 5 CDSs and 20 exons. This estimate is very close to the predicted exon/gene ratio of 4.245, based on the regression model, when the average exon length was the predictor or 3.658 when the median exon length was used (Table 2). The predicted values based on the average exon length for the other two higher plants analyzed were also close to the observed values (5.970 vs. 5.148 in thale cress and 5.654 vs. 4.790 in rice). The observed values for all three species fell inside the 95% CI at the individual level.

The number of protein coding genes and total number of genes expected in an organism with the average exon length of 334.8 bp (such as in *P. taeda*) is 13,871 and 13,288, respectively. These values seem to be underestimated as far as *P. taeda* is concerned, especially when compared with the other analyzed plants. Moreover, there are currently about nineteen thousand unique sequences in the NCBI UniGene database for *P. taeda*. The model severely underestimates the number of genes in the other two vascular plants described above as well. The number of protein coding genes in *A. thaliana* is underestimated by about 34.4% and *O. sativa* by about 36.9%. If *P. taeda* followed this bias, and the expected number of protein coding genes was also underestimated by approximately 35%; that would mean about 7,155 underestimated genes, which would raise the predicted number of protein coding genes to about 20,443 in this species, making this number more realistic.

Regression models that are based exclusively on the three examined plants and that follow the same logic as in the case of the 36 studied genomes also demonstrated that higher numbers are expected for loblolly pine (Table 2). The total number of genes expected would be 20,923 (upper limit is 45,975 at 95% confidence level; model significant at 93.7% confidence level) and the number of protein coding genes 19,785 (upper limit is 32,508 at 95% confidence level). These numbers seem to be more realistic when compared to the observed values in other higher plants, especially considering broad confidence intervals. Although the above findings for loblolly pine are restricted by the limited availability of the data, this study is the first attempt to address the questions of exon-intron structure and genomic complexity in this species, and will likely stimulate further studies.

### Conclusions

This study confirmed the general trend of increasing number of genes, gene products, and exons in the genome, along with higher exon/gene ratio and AS ratio as species become more evolutionarily advanced. We demonstrated that parameters easily computable from small data samples (e.g. exon length or exon/gene ratio) are relatively good predictors of characteristics that are difficult to assess, such as total number of genes, gene products and exons. We also showed that taxonomic kingdoms may require different model calibration as their strategies to increase complexity throughout evolution have been different. As more genomic data become available and more species representing various taxonomic groups are annotated, these models can be tuned or applied to specific monophyletic groups, which will improve precision of the predictions.

## Materials and Methods

### Selection of completely sequenced species for analysis

We selected the 36 most-annotated and featured species (Table 1) from eukaryotic genomic assemblies available in the NCBI GenBank [41]. For 10 species in our set (*Arabidopsis thaliana*, *Caenorhabditis elegans*, *Drosophila melanogaster*, *Encephalitozoon cuniculi*, *Eremothecium gossypii*, *Homo sapiens*, *Mus musculus*, *Oryza sativa*, *Saccharomyces cerevisiae* and *Schizosaccharomyces pombe*), the annotation involved at least partial curation of the records. AS in rice was not documented in the present NCBI GenBank genome annotation, although it has been reported previously [23], [24]. Therefore, this species was excluded from the AS analysis.

### Source of data

All genomic data were downloaded from the FTP directory of the NCBI GenBank (ftp://ftp.ncbi.nih.gov/genomes/MapView/). Sequences for *P. taeda* were downloaded from the Nucleotide database from the NCBI GenBank.

### Genomic data analysis

Genomic data were analyzed using Perl scripts specifically written for this study (available at http://treenome.tamu.edu/). The downloaded files were screened, and chromosome ID, position and orientation of the exons on the chromosome, feature ID, AS type, transcript accession number, and group label were traced, partitioned and analyzed. Pseudogenes, mitochondrial, plastid and insufficiently annotated genes were excluded from further analysis. Total numbers of genes, protein coding genes and their coding sequences (CDSs) were calculated for each species. The number of exons and their boundaries were determined based on the coding structure of each protein coding gene ID recorded in their corresponding CDS section. For each gene supported by more than one CDS, the alternative coding sequences were compared with each other. Cases when corresponding exons had different boundaries or no matching counterpart were qualified as AS variants. Average and median lengths were calculated for both exons and CDSs. The exon estimates were computed based on all unique exons found in the genome. All CDSs, including alternatively spliced forms, were considered for estimation of the average and median CDS lengths. The exon/gene ratio was defined as the average number of exons per protein coding gene. AS ratio was defined as the ratio between the number of alternatively spliced and all protein coding genes. AS variants were categorized using the binary approach described by Nagasaki et al. [42]. Shannon's index *H* and equitability *E* were calculated for genes with AS to reflect richness and distribution evenness of AS forms [43].

### Parameter estimation for *Pinus taeda*

In total, 99 complete CDS sequences representing protein coding genes in *P. taeda* were downloaded from NCBI GenBank, and their CDS structure and length were analyzed. The data were prescreened by a Perl script and rearranged manually. The majority of these sequences represented mRNA/cDNA. Only five CDSs represented genomic sequences and could provide complete information about exon-intron structure. Average and median CDS and exon lengths were calculated based on this information. Using the mean exon length and regression models developed based on the genomic data for other species, we computed the expected total number of exons, total number of genes, exon/gene ratio, and total number of protein coding genes for *P. taeda*. Software package JMP version 5 was used for the statistical analysis.

## Supporting Information

Figure S1 **Correlation of number of all genes and mean exon length in the genomes of 36 species.**
(TIF)

Figure S2 **Correlation of mean CDS length and mean exon length in the genomes of 36 species.**
(TIF)

Figure S3 **Correlation of number of all genes and number of all exons in the genomes of 36 species.**
(TIF)

Figure S4 **Correlation of number of protein coding genes and exon/gene ratio in the genomes of 36 species.**
(TIF)

Figure S5 **Correlation of alternative splicing ratio and mean exon length.** Only 12 species with alternative splicing were considered.
(TIF)

Figure S6 **Correlation of alternative splicing ratio and exon/gene ratio.** Only 12 species with alternative splicing were considered.
(TIF)

Figure S7 **Correlation of alternative splicing ratio and number of all CDSs.** Only 12 species with alternative splicing were considered.
(TIF)

Table S1 **Exon-intron gene structure in completely sequenced genomes of 56 species.**
(XLS)

Table S2 **Regression models.**
(XLS)
