# Supplementary figures and images for: Evolution of Exon-Intron Structure and Alternative Splicing
Source: PLoS One. 2011 Mar 25;6(3):e18055. doi: 10.1371/journal.pone.0018055 (PMC3064661; doi:10.1371/journal.pone.0018055)

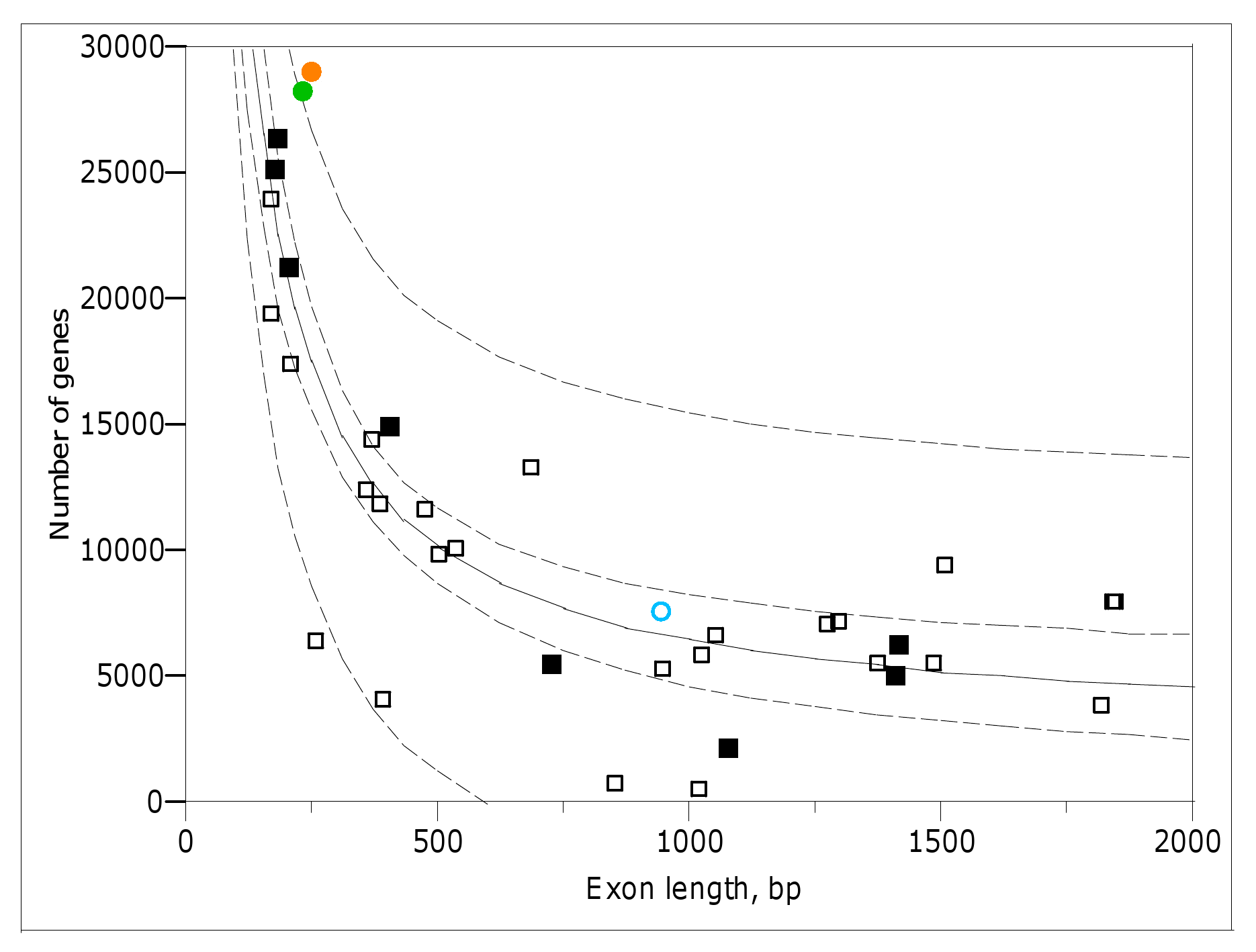

Supplement: Figure S1 — Correlation of number of all genes and mean exon length in the genomes of 36 species. (TIF) [file pone.0018055.s001.tif]

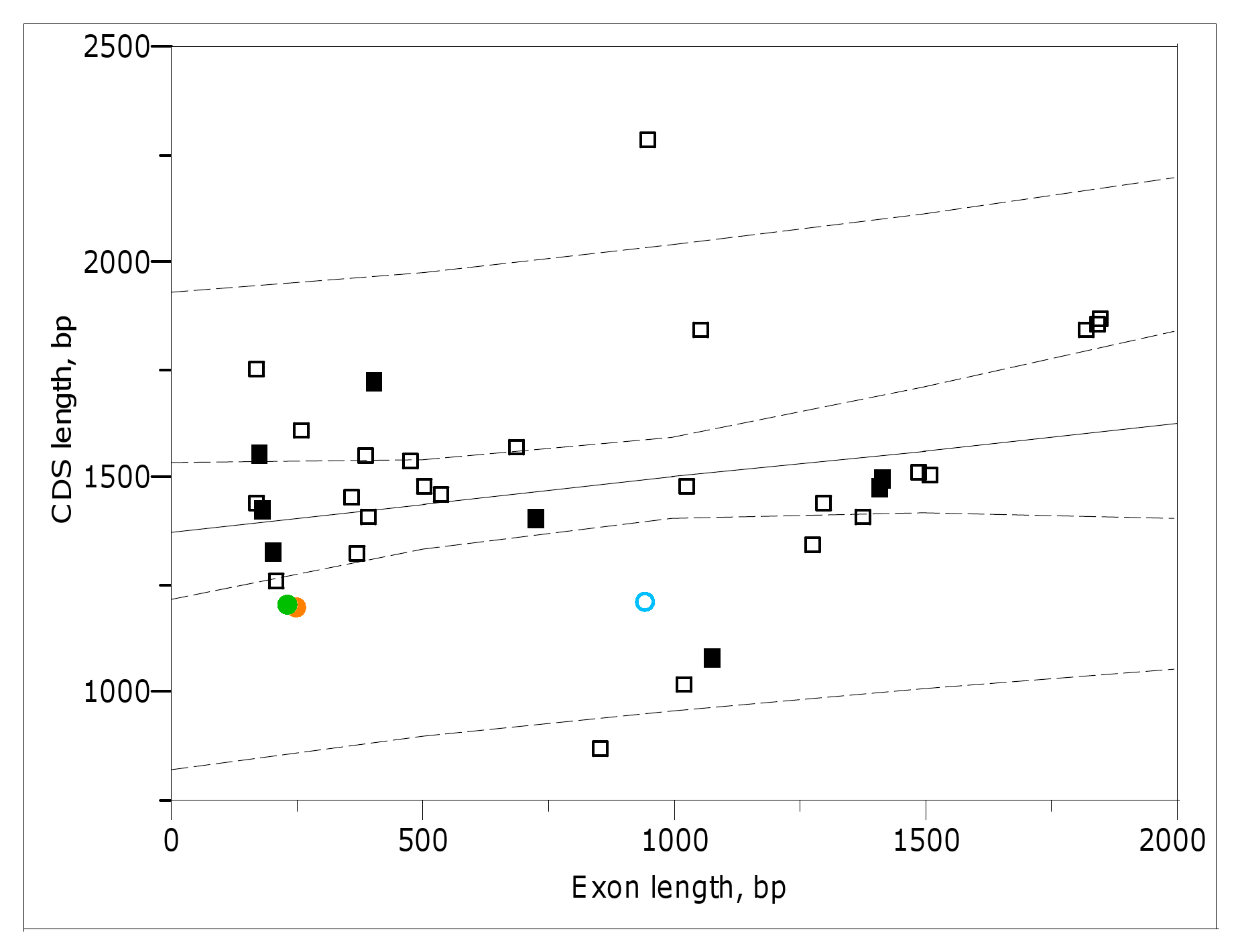

Supplement: Figure S2 — Correlation of mean CDS length and mean exon length in the genomes of 36 species. (TIF) [file pone.0018055.s002.tif]

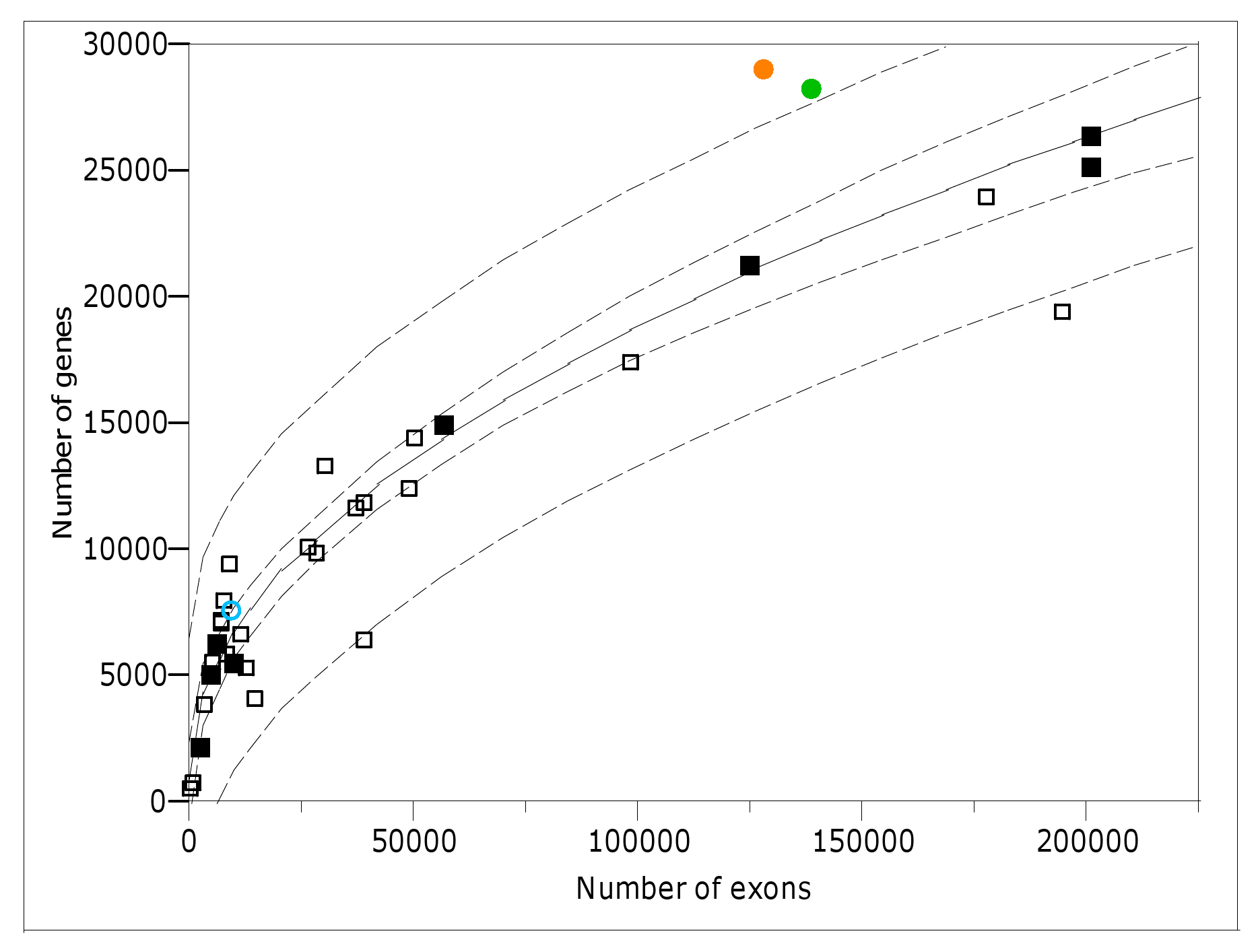

Supplement: Figure S3 — Correlation of number of all genes and number of all exons in the genomes of 36 species. (TIF) [file pone.0018055.s003.tif]

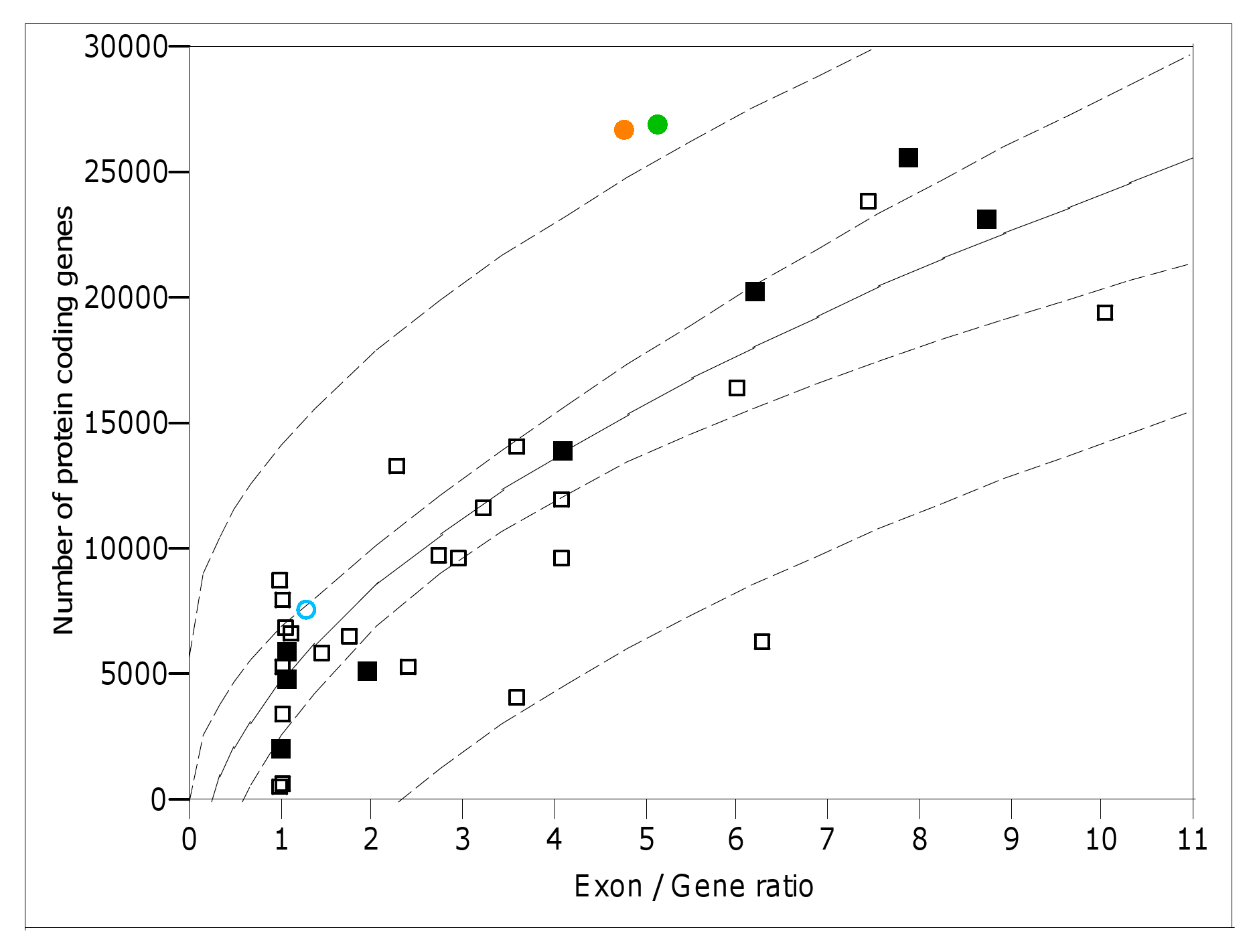

Supplement: Figure S4 — Correlation of number of protein coding genes and exon/gene ratio in the genomes of 36 species. (TIF) [file pone.0018055.s004.tif]

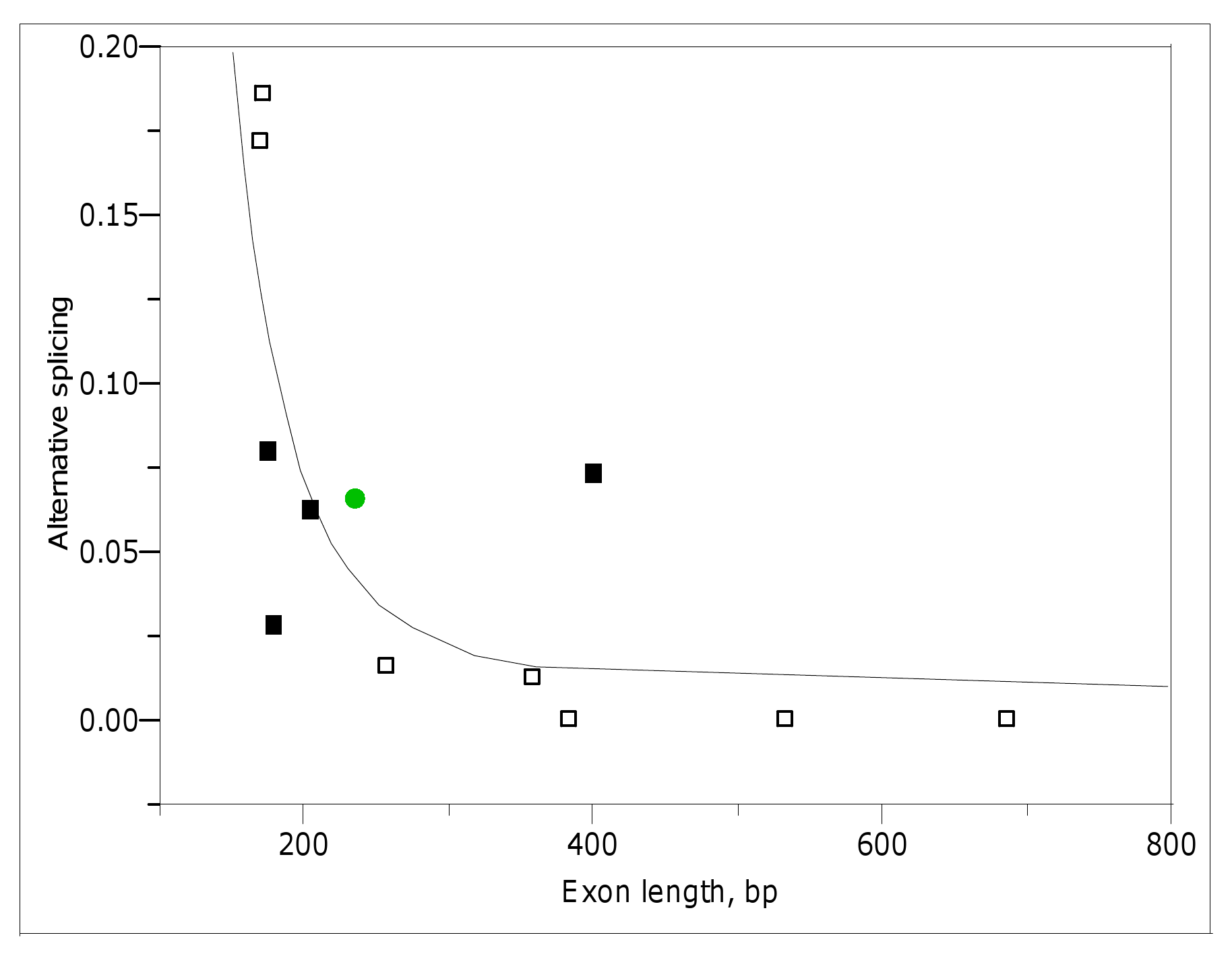

Supplement: Figure S5 — Correlation of alternative splicing ratio and mean exon length. Only 12 species with alternative splicing were considered. (TIF) [file pone.0018055.s005.tif]

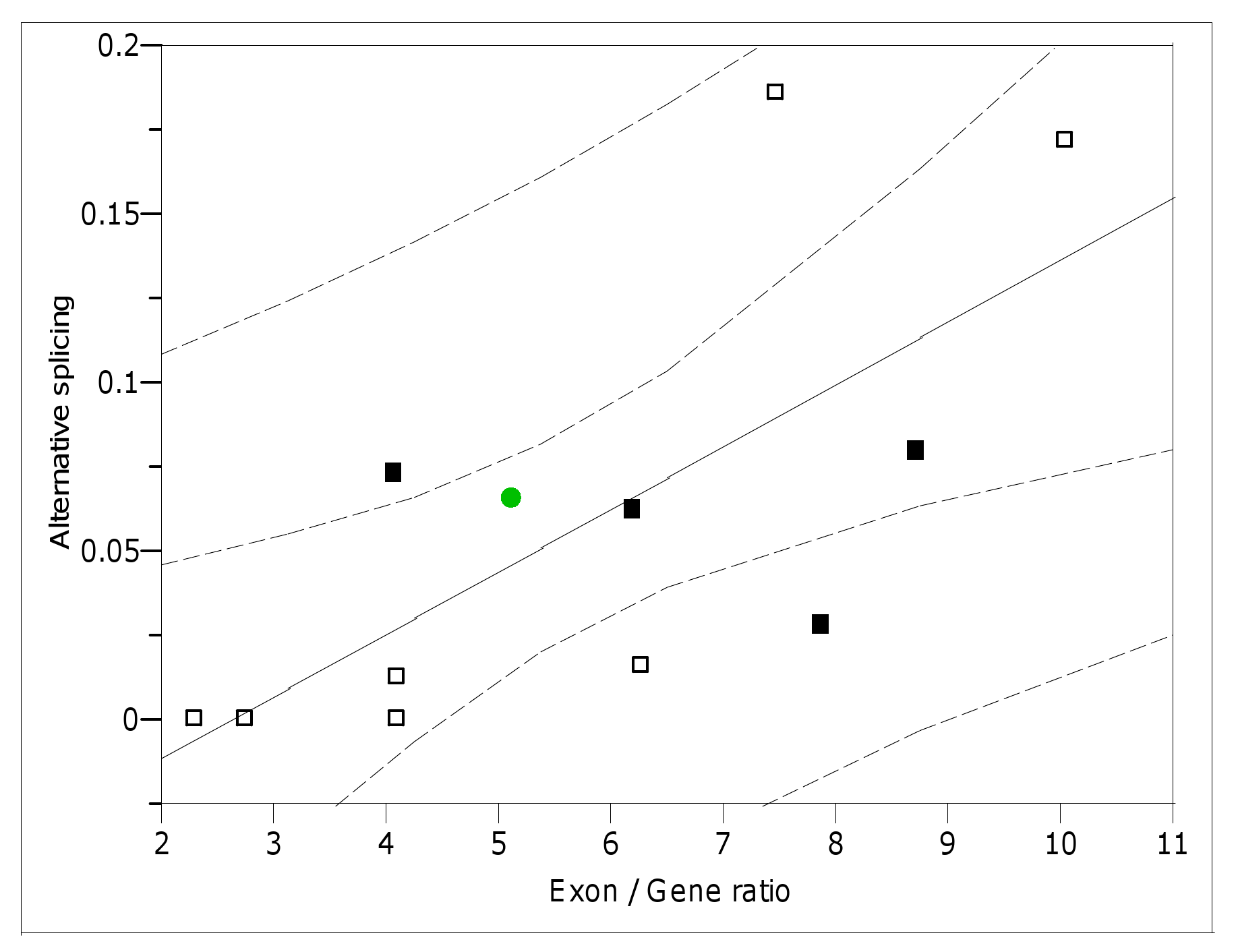

Supplement: Figure S6 — Correlation of alternative splicing ratio and exon/gene ratio. Only 12 species with alternative splicing were considered. (TIF) [file pone.0018055.s006.tif]

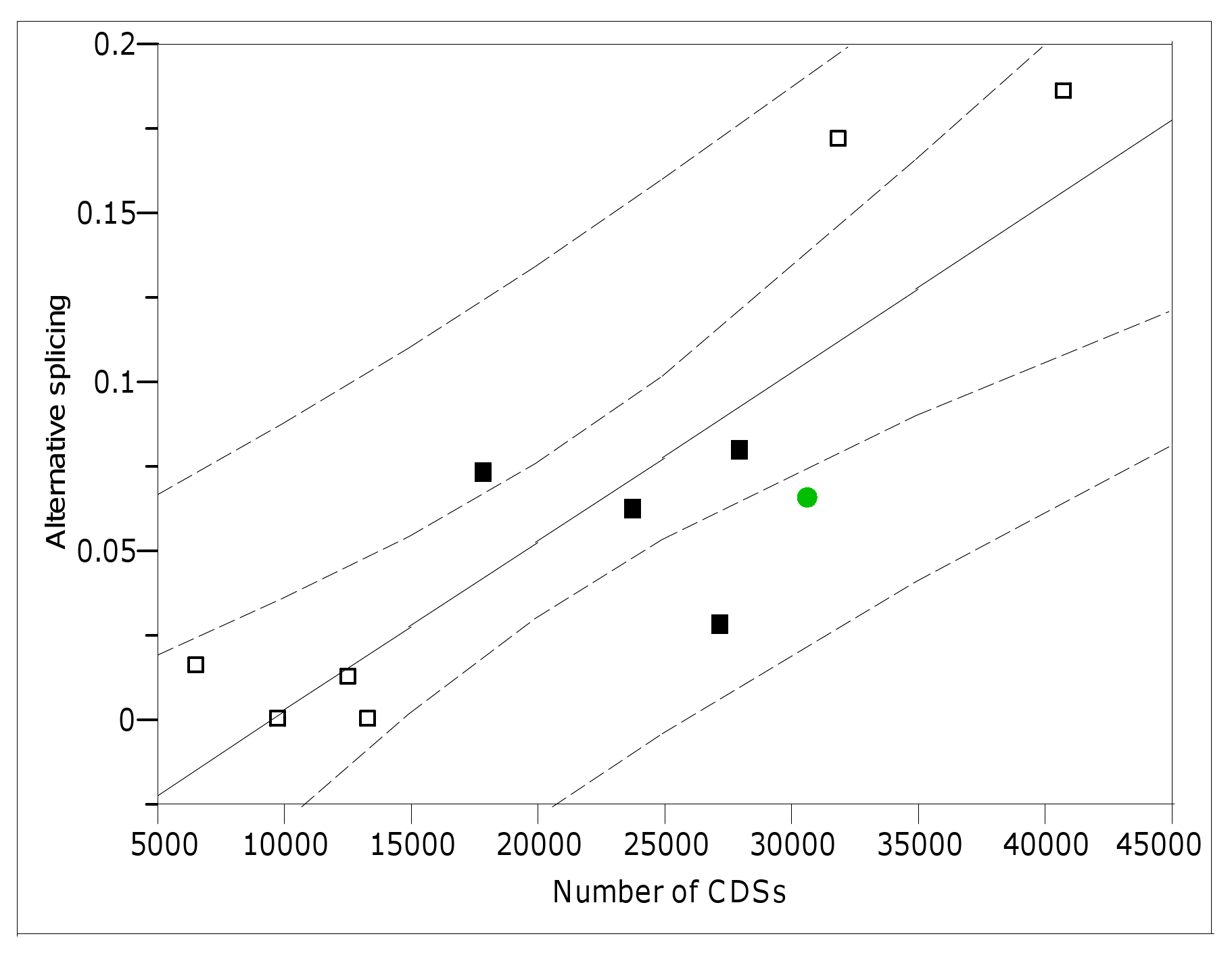

Supplement: Figure S7 — Correlation of alternative splicing ratio and number of all CDSs. Only 12 species with alternative splicing were considered. (TIF) [file pone.0018055.s007.tif]
